# *p, p′*-Dichlorodiphenyldichloroethylene Induces Colorectal Adenocarcinoma Cell Proliferation through Oxidative Stress

**DOI:** 10.1371/journal.pone.0112700

**Published:** 2014-11-11

**Authors:** Li Song, Jianxin Liu, Xiaoting Jin, Zhuoyu Li, Meirong Zhao, Weiping Liu

**Affiliations:** 1 Institute of Biotechnology, Key Laboratory of Chemical Biology and Molecular Engineering of National Ministry of Education, Shanxi University, Taiyuan, China; 2 MOE Key Lab of Environmental Remediation and Ecosystem Health, College of Environmental and Resource Sciences, Zhejiang University, Hangzhou, China; 3 College of Life Science, Zhejiang Chinese Medical University, Hangzhou, China; 4 Research Center of Environmental Science, Zhejiang University of Technology, Hangzhou, China; University of Kentucky, United States of America

## Abstract

*p, p′*-Dichlorodiphenyldichloroethylene (DDE), the major metabolite of Dichlorodiphenyltrichloroethane (DDT), is an organochlorine pollutant and associated with cancer progression. The present study investigated the possible effects of *p,p′*-DDE on colorectal cancer and the involved molecular mechanism. The results indicated that exposure to low concentrations of *p,p′*-DDE from 10^−10^ to 10^−7 ^M for 96 h markedly enhanced proliferations of human colorectal adenocarcinoma cell lines. Moreover, *p,p′*-DDE exposure could activate Wnt/β-catenin and Hedgehog/Gli1 signaling cascades, and the expression level of c-Myc and cyclin D1 was significantly increased. Consistently, *p,p′*-DDE-induced cell proliferation along with upregulated c-Myc and cyclin D1 were impeded by β-catenin siRNA or Gli1 siRNA. In addition, *p,p′*-DDE was able to activate NADPH oxidase, generate reactive oxygen species (ROS) and reduce GSH content, superoxide dismutase (SOD) and calatase (CAT) activities. Treatment with antioxidants prevented *p,p′*-DDE-induced cell proliferation and signaling pathways of Wnt/β-catenin and Hedgehog/Gli1. These results indicated that *p,p′*-DDE promoted colorectal cancer cell proliferation through Wnt/β-catenin and Hedgehog/Gli1 signalings mediated by oxidative stress. The finding suggests an association between *p,p′*-DDE exposure and the risk of colorectal cancer progression.

## Introduction

Dichlorodiphenyltrichloroethane (DDT) was the most used organochlorine pesticide in the world. DDT became banned in the 1970s in most western countries because of its adverse effects on wildlife. DDT is still being used to prevent malaria and typhoid in some developing countries [1], [2]. Although DDT has generally been restricted for use over decades, the metabolites exposure still exists mainly through metabolic conversion in the body. *p, p′*-Dichlorodiphenyldichloroethylene (*p, p′*-DDE) is DDT’s major metabolite with high persistence and lipophilicity. High *p, p′*-DDE levels are usually found in human blood and tissues [3]–[6]. Accumulated evidences indicate that *p, p′*-DDE exposure is related to repercussions in human health, such as neurotoxicity, immunotoxicity and cancinogenesis. [7]–[13]. Thus, investigations involved in adverse effects of *p, p′*-DDE on human health, especially its link with cancer, are receiving more and more attentions.

Colorectal cancer (CRC) is the third most commonly diagnosed cancer and the second leading cause of cancer death in the United States [14]. The CRC incidence is related to multiple risk factors including hereditary factor, lifestyle and body mass [15]–[17]. It is noteworthy that chemical contamination of food is also considered as an potential factor, which has increased the concerns of the public health [18]–[24].

Epidemiological investigations have suggested an association between *p,p′-*DDE exposure and CRC. Higher serum *p,p′-*DDE levels were found in CRC patients from Egypt [19], [20]. In China, CRC incidence was significantly related to residual *p,p′-*DDE in rice [18]. Howsam *et al* discovered that the role of *p,p′*-DDE in CRC risk may be more complex [24]. The association between *p,p′*-DDE exposure and CRC was not remarkable for entire population of cases, but they were significant for the subset of tumors with mutation of *p53* gene or wild-type K-*ras* [24]. Although epidemiological data suggested *p,p′*-DDE exposure may improving CRC incidence, the molecular mechanism underlying its promoting effects on CRC remains unclear.

Aberrant cell signaling pathways play vital roles in most CRCs progression. Both Wnt/β-catenin and Hedgehog/Gli1 signaling pathways were highly implicated in CRC development [25]–[28]. In the presence of Wnt signaling, β-catenin is released from a destruction complex including Axin, tumor suppressors adenomatous polyposis (APC), glycogen synthase kinase-3β (GSK3β) and casein kinase 1α (CK1α). β-catenin becomes stable in cytoplasm and then transported into cell nucleus to regulate target genes transcription [29]. When Hedgehog/Gli1 pathway is activated, the binding of Hh ligand to PTCH1 protein relieves the SMO signal transducer from Patched-dependent suppression. Gli1 is accumulated in the nuclear, and leads to Gli1-dependent transcriptional activation of targets genes [30]–[32]. Activation of both Wnt/β-catenin and Hedgehog/Gli1 signaling pathways results in the over-expression of cancer-related genes such as *cyclin D* and *Myc*, which are involved in cancer development [29], [32], [33].

It has been known that *p,p′*-DDE could induce the generation of reactive oxygen species (ROS) such as superoxide (O_2_^−^) and hydroxyl radical (OH^−^). Shi *et al*. [34] showed *p,p′*-DDE induced apoptosis of rat sertoli cells by ROS dependent activation of NF-κB and FasL-dependent pathways. Oxidative stress plays critical role in inducing CRC progression. Some carcinogens such as arsenic and chromium promote CRC development via ROS-mediated pathways [35], [36]. Our previous study demonstrated that *p,p′*-DDT, precursor of *p,p′*-DDE induced colorectal cancer growth through oxidative stress-mediated pathways [37]. Therefore, it is likely that *p,p′*-DDE promotes CRC by oxidative stress-mediated signaling pathways.

The aim of the present study is to investigate the effects of low concentrations of *p,p′*-DDE on colorectal cancer and the involved mechanism concerning oxidative stress and two critical CRC–related pathways, Wnt/β-catenin and Hedgehog/Gli1 signalings. The results demonstrated that *p,p′*-DDE exposure promoted colorectal adenocarcinoma cell proliferation through activated Wnt/β-catenin and Hedgehog/Gli1 signalings, which were mediated by oxidative stress.

## Materials and Methods

### Chemicals and reagents

*p, p′*-Dichlorodiphenyldichloroethylene (*p, p′*-DDE) was from Bestown (Beijing, China) and was dissolved in dimethyl sulfoxide (DMSO, Sigma) to get the stock concentration of 100 mM for the *in vitro* assays. 3-(4, 5-dimethylthiazol-2-yl)-2, 5-diphenyl tetrazolium bromide (MTT), superoxide dismutase (SOD) and catalase (CAT) were purchased from Sigma (St. Louis, MO, USA). *N-Acetyl-L-cysteine* (NAC), 2′, 7′-dichlorofluorescein diacetate (DCFH-DA), cytoplasmic and nuclear protein extraction kit, BCA protein kit and western lysis buffer were from Beyotime Institute of Biotechnology (Nan tong, China). RPMI 1640 medium, Dulbecco’s modified Eagle medium (DMEM) and fetal bovine serum were from Gibco BRL (Grand Island, NY, USA). Assay kits for superoxide dismutase (SOD), glutathione (GSH) and catalase (CAT) were provided by the Nanjing Jiancheng Corp (China). Enhanced chemiluminescence kit was from Peirce (Tottenhall, UK). Trizol reagent, PrimeScript RT Master Mix and the SYBR green-based RT-PCR kit were from Takara Biotechnology (Japanese). Antibody to GSK3β and phospho-GSK3β (Ser9) were obtained from Cell Signaling (Beverly, MA, USA). Antibody to β-catenin and PCNA were obtained from Abmart (USA). Antibody to phospho-β-catenin (Ser33) was from BBI (UK). Antibodies to c-Myc, Lamin B1 and cyclin D1 were purchased from Bioword (USA). Antibody to α-tubulin was a gift from Professor Inke S. Nathke (University of Dundee, UK). Antibody to Gli1 was purchased from Santa Cruz (Heidelberg, Germany). Horseradish peroxidase (HRP)-linked anti-rabbit IgG, anti-mouse IgG, anti-rat IgG and Lipofectamine 2000 were purchased from Invitrogen (USA). Non-silencing small interference RNA (siRNA), β-catenin-siRNA and Gli1-siRNA were synthesized by Genepharma Company (Shanghai, China).

### Cell culture and treatment

Human colorectal adenocarcinoma DLD1 and SW620 cells were purchased from the Institute of Cell Research (Shanghai, China) and cultured as described previously [37], [38]. To observation of *p,p′*-DDE cytotoxicity, cells were exposed to various concentrations of *p,p′*-DDE (from 10^−12^ to 10^−4 ^M) for 96 h. Control group was 0.1% (v/v) DMSO in medium. To detect the effect of β-catenin or Gli1 silence on *p,p′*-DDE-induced DLD1 cell proliferation, cells were transfected with β-catenin, Gli1 siRNA or non-silencing siRNA, followed by incubating in medium containing 10^−9 ^M of *p,p′*-DDE or the same amount of DMSO as the working solution. To determine the effects of NAC, SOD or CAT on *p,p′*-DDE-induced colorectal adenocarcinoma cell proliferation, cells were exposed to 10^−9 ^M of *p,p′*-DDE alone or co-exposed with NAC (10^−3 ^M), SOD (100 U/ml) or CAT (500 U/ml) for 96 h. The equal amount of DMSO was added to medium for control cells.

### Cell viability assay and cell counts

Cell viability was determined by MTT assay. Cells were seeded in 96-well plates (10^3^ cells per well). After treatment with *p,p′*-DDE for 96 h, cells were incubated in culture medium containing MTT (0.5 mg ml^−1^) for 4 h at 37°C. The medium was replaced by DMSO and the absorbance at 570 nm was measured using a microplate reader. Cell viability (%) was defined relative to untreated control and calculated based on the following formula: Viability (% above control) = (A_570_ of treated cells - A_570_ of control)/A_570_ of control×100. Meanwhile, cells were seeded in 12-well plates (5×10^4^ cells per well) and exposure to *p,p′*-DDE for 96 h. Total number of viable cells was counted by trypan blue exclusion method using a hemocytometer. Proliferation rate (% above control) = (Number of treated cells – Number of control cells)/number of control cells×100. When cells were exposed to high concentrations of *p,p′*-DDE at 10^−5^ and 10^−4 ^M, inhibition rate (%) in MTT assay was calculated based on the following formula: inhibition rate (%) = (A_570_ of control – A_570_ of treated cells)/A_570_ of control×100. In cell counts, inhibition rate (%) = (Number of control cells – Number of treated cells)/number of control cells×100.

### RNA isolation and Quantitative real-time PCR analysis

Cells were incubated with various concentrations of *p,p′*-DDE (10^−10^, 10^−11^, 10^−9 ^M) for 96 h. In antioxidants experiments, cells were cultured in 10^−9 ^M of *p,p′*-DDE alone or co-cultured with NAC (10^−3 ^M), SOD (100 U/ml) or CAT (500 U/ml) for 96 h. Total RNA was extracted from treated cells using trizol reagent. 1µg of DNA-free total RNAs was reverse transcribed using PrimeScript RT Master Mix. For RT-PCR assay, 25 µL reaction mixture containing 4 µL cDNA, 1 µL primers and 12.5 µL SYBR Premix Ex Taq were used to detect double-stand DNA synthesis. The forward (F) and reverse (R) primers were as follows: PTCH1 (F): 5′-ACCAGAATGGGTCCACGACAA-3′; PTCH1 (R): 5′-AAAGTCTGAGGTGTCCCGCAA-3′; Nox1 (F): 5′-CACAAGAAAAATCCTTGGGTCAA-3′; Nox1 (R): 5′-GACAGCAGATTGCGACACACA-3′; p22^phox^ (F): 5′-CGCTGGCGTCCGCCTGATCCTCA-3′; p22^phox^ (R): 5′-ACGCACAGCCGCCAGTAGGTAGAT-3′; p40^phox^ (F): 5′-TGAACAGCTTCCGGATGATG-3′; p40^phox^ (R): 5′-TGAAGCCTCTCTTCTCCTCGAT-3′; p47^phox^ (F): 5′-GTCAGATGAAAGCAAAGCGA-3′; p47^phox^ (R): 5′-CATAGTTGGGCTCAGGGTCT-3′; p67^phox^ (F): 5′-ATCAGCCTCTGGAATGAAGGGG-3′; p67^phox^ (R): 5′-GCAGCCAATGTTGAAGCAAATCC-3′; GAPDH (F): 5′-GCACCGTCAAGGCTGAGAAC-3′; GAPDH (R): 5′-GCACCGTCAAGGCTGAGAAC-3′.

### Protein extraction and Western blotting

Treated cells were lysed using western lysis buffer for 10 min, followed by centrifugation at 13,000×g for 15 min to precipitate insoluble material. Cytoplasmic and nuclear proteins were extracted according the instructions of cytoplasmic and nuclear protein extraction kit. Protein concentration was measured using a BCA protein kit. 40 µg of cell lysates were separated by 12% SDS-PAGE and transferred to nitrocellulose membranes using a BIO-RAD mini-transblot system. Membranes were blocked for 1 h in Tris-buffered saline (TBS) containing 5% milk and Tween 20, followed by incubation with anti-β-catenin (1∶1000), anti-phospho-GSK3β (Ser9) (1∶1000), anti-phospho-β-catenin (Ser33) (1∶500), anti-Gli1 (1∶500), anti-c-Myc (1∶500), anti-Lamin B1 (1∶500), anti-cyclin D1 (1∶500), anti-PCNA(1∶1000) or anti-α-tubulin (1∶500) overnight at 4°C. Following three washes in TBS, membranes were incubated with secondary antibodies (1∶1000) for 1 h at room temperature and incubated in chemiluminescence detection substrate for 5 min followed by X-ray film exposure.

### RNA interference

DLD1 cells grown in 6-well plates (10^6^ cells per well) or in 96-well plates (10^3^ cells per well) were transfected with 100 nM β-catenin siRNA, Gli1 siRNA or non-silencing siRNA using Lipofectamine 2000 according to manufacturer’s protocol. After transfection for 6 h, cells were treated with 10^−9 ^M of *p,p′*-DDE for 96 h, followed by performing cell viability and western blot assay. The following siRNA were used:

β-catenin siRNA: 5′-CAGUUGUGGUUAAGCUCUUdTdT-3′;

Gli1 siRNA: 5′-CUCCACAGGCAUACAGGAUTT;

Non-silencing siRNA: 5′- TTCTCCGAACGTGTCACGT-3′.

### Measurement of intracellular ROS, GSH content, SOD and CAT activities

After cells were treated with *p,p′*-DDE (10^−9 ^M) with or without NAC (10^−3 ^M), SOD (100 U/ml) or CAT (500 U/ml) for 96 h, reactive oxygen species (ROS), GSH content, superoxide dismutase (SOD) and catalase (CAT) activities were evaluated using commercial assay kits. For ROS measurements, cells were incubated with 10 µM of 2′, 7′-dichlorofluorescein diacetate (DCFH-DA) at 37°C for 30 min. Cells were collected and the production of ROS was detected by changes in fluorescence due to the accumulation of DCF using a fluorescence microplate reader (Thermo Scientific varioskan flash). For GSH content, SOD and CAT activities, cells were suspended in physiological saline (PBS), followed by ultrasound treatment and centrifugation at 6,000 rpm for 10 min. The supernatant of cell lysates were used for experiments. GSH levels were determined at 420 nm using 5, 5′-dithiobis-2-nitobenzoic acid. SOD activity was examined for reduction of nitroblue tetrazolium (NBT). CAT activity was determined by the decomposition of H_2_O_2_.

### Statistical analysis

All data were presented as means ± standard deviation from three independent experiments. Statistical analyses used the SPSS 10 software. Data were analyzed by Student’s *t*-test where two treatments were compared or one-way analysis of variance (ANOVA) with Tukey’s multiple comparison tests for comparisons of more than two treatments. Statistically significant was defined as *p*<0.05.

## Results

### Low concentrations of *p,p′*-DDE promote colorectal adenocarcinoma cell proliferation

**Figure 1:**
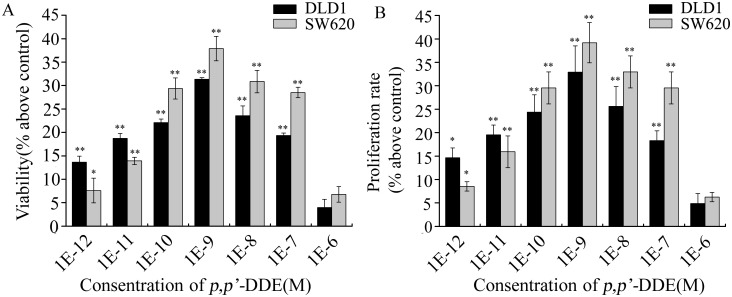
Low concentrations of *p,p′*-DDE promote colorectal adenocarcinoma cell proliferation. After DLD1 or SW620 cells were exposed to 10^−12^ to 10^−6 ^M *p,p′*-DDE for 96 h, cell viability (A) and proliferation rate (B) were determined using MTT and cell number assays, respectively. Values are percent changes above the control (DMSO, 0.1%) as the mean ± SD of three independent experiments. *p<0.05 and **p<0.01 compared to control cells.

To test the effects of *p,p′*-DDE on colorectal adenocarcinoma cells, cell viability and viable cell counts were analyzed after DLD1 or SW620 cells treated with *p,p′-*DDE (10^−12^ to 10^−4 ^M) for 96 h. MTT and cell count assays showed that *p,p′*-DDE exposure at 10^−4 ^M significantly inhibited cell viability and decreased viable cell counts in both DLD1 and SW620 cells (Fig. S1). However, at 10^−5^ and 10^−6 ^M no significant effects were observed (Fig. S1 and 1). When colorectal adenocarcinoma cells were exposed to lower concentrations of *p,p′*-DDE (10^−12^ to 10^−7 ^M), cell viability and viable cell counts were significantly increased (Fig. 1). Treatment with 10^−9 ^M *p,p′*-DDE resulted in the biggest increase in cell viability and viable cell counts. Taken together, these results indicated that low concentrations of *p,p′-*DDE (10^−12^ to 10^−7 ^M) markedly promote colorectal adenocarcinoma cell proliferation.

### *p,p′*-DDE stimulates both Wnt/β-catenin and Hedgehog/Gli1 signalings in colorectal adenocarcinoma cells

**Figure 2:**
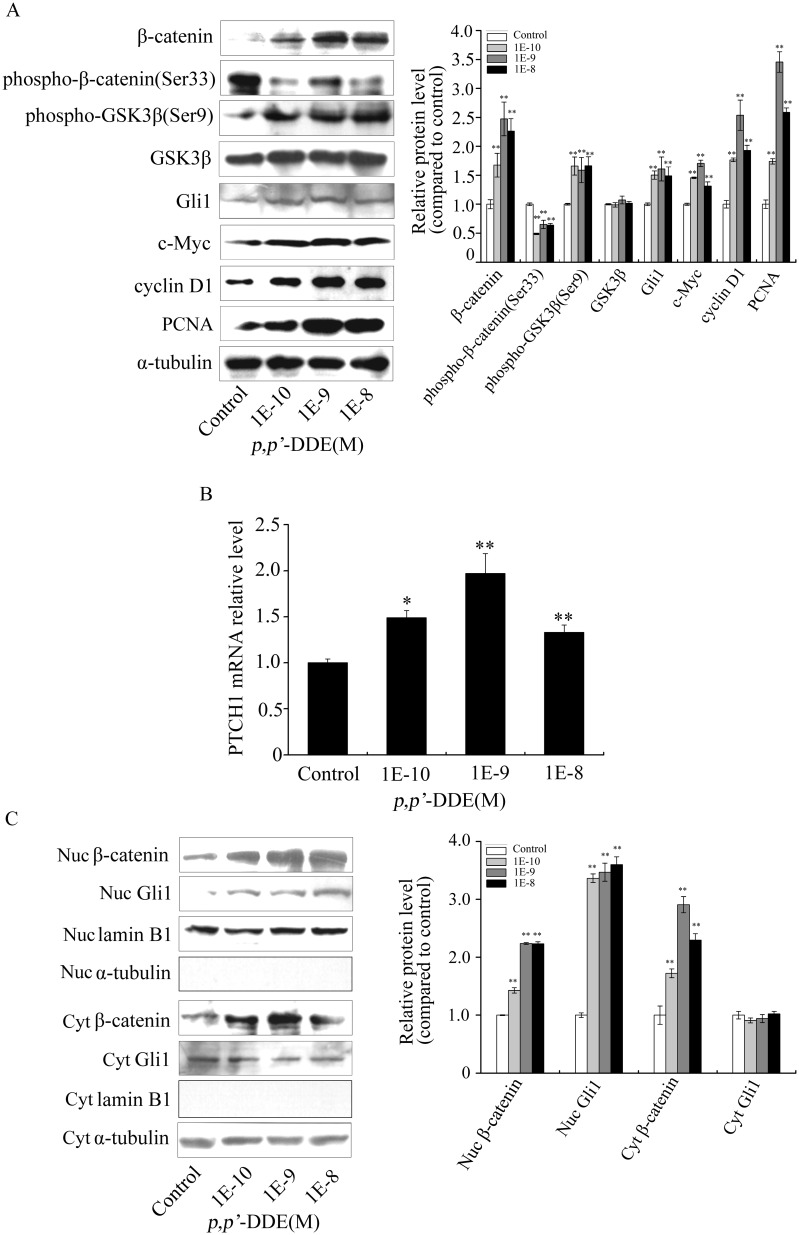
*p,p′*-DDE upregulates Wnt/β-catenin and Hedgehog/Gli1 signalings in DLD1 cells. After DLD1 cells were treated with *p,p′*-DDE (10^−10^, 10^−9^, 10^−8 ^M) for 96 h, (A) the protein levels of β-catenin, phospho-β-catenin (Ser33), phospho-GSK3β (Ser9), GSK3β, Gli1, c-Myc, cyclin D1 and PCNA were examined by western blotting. α-tubulin was used as the loading control; Grayscale scan analysis of western blot bands from three independent experiments were shown in right panel of graph. **p<0.01 compared to control cells. (B) mRNA expression of PTCH1 was quantified by quantitative real-time PCR analysis. Relative mRNA levels were normalized with control mRNA. The data were acquired from three biologically independent experiments. Value shown was given as the ± SD. *p<0.05 and **p<0.01 compared to control. (C) Western blotting was performed to analyze the amounts of cytoplasmic or nuclear β-catenin and Gli1 proteins. α-tubulin or lamin B1 was used as a control. Grayscale scan analysis of western blot bands from three independent experiments were shown in right panel of graph. **p<0.01 compared to control cells.

Both Wnt/β-catenin and Hedgehog/Gli1 signalings are abnormal activated in colorectal cancer [25]–[28]. To elucidate the mechanism of *p,p′*-DDE in promoting colorectal adenocarcinoma cell proliferation, both Wnt/β-catenin and Hedgehog/Gli1 signalings were examined after cells treated with *p,p′*-DDE (10^−10^, 10^−9^ and 10^−8 ^M) for 96 h. As shown in Fig. 2A and S2A, *p,p′*-DDE treatment resulted in increased β-catenin and phospho-GSK3β (Ser9) levels along with decreased phospho-β-catenin (Ser33) levels in DLD1 or SW620 cells. It suggested that *p,p′*-DDE stimulated Wnt/β-catenin signaling in colorectal adenocarcinoma cells. In addition, *p,p′*-DDE exposure increased Gli1 protein level and mRNA level of PATCH1 (Fig. 2A, 2B and S2), which suggested *p,p′*-DDE could stimulate Hedgehog/Gli1 signaling. β-catenin or Gli1, acting as nuclear executors of Wnt/β-catenin or Hedgehog/Gli1 signaling, inputs cell nuclear and induces target gene transcription [25]. Therefore, the intracellular distribution of β-catenin or Gli1 after DLD1 cells exposed to *p,p′*-DDE was examined. The data indicated that *p,p′*-DDE exposure led to the accumulation of both β-catenin and Gli1 in the nucleus (Fig. 2C).

Activation of Wnt/β-catenin and Hedgehog/Gli1 signalings results in overexpression of downstream c-Myc and cyclin D1, which are involved in cancer cell proliferation [29], [32], [33]. Therefore, c-Myc, cyclin D1 and downstream proliferation effector PCNA were examined after *p,p′*-DDE treatment. The results showed that c-Myc, cyclin D1 and PCNA were up regulated in *p,p′-*DDE-treated cells (Fig. 2A and S2A). These results suggested that overexpression of both c-Myc and cyclin D1 were involved in *p,p′*-DDE-induced colorectal adenocarcinoma cell proliferation.

### Blockage of Wnt/β-catenin or Hedgehog/Gli1 signaling prevents *p,p′*-DDE-induced colorectal adenocarcinoma cell proliferation

**Figure 3:**
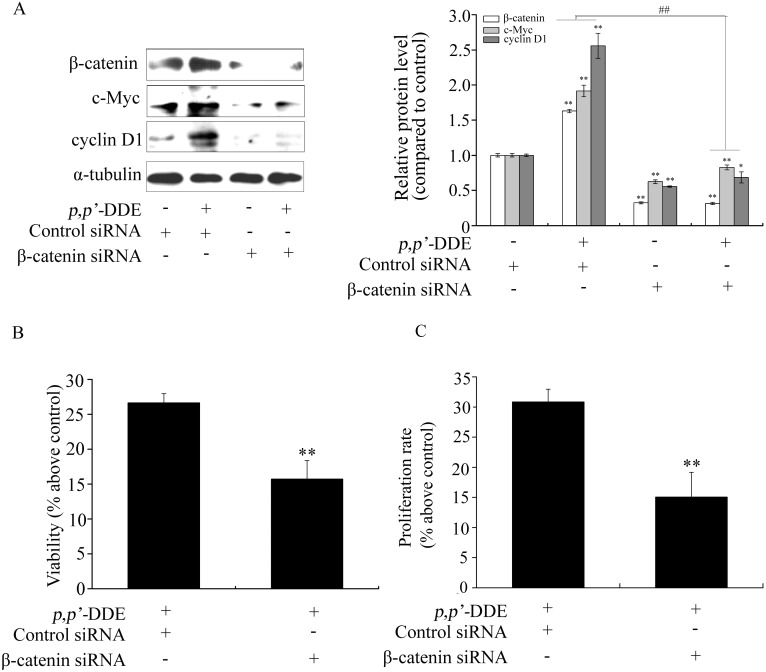
Inhibition of Wnt/β-catenin signaling blocks *p,p′*-DDE-induced DLD1 cell proliferation. After DLD1 cells were transfected with control siRNA or β-catenin siRNA, followed by treating with *p,p′*-DDE (10^−9 ^M) for 96 h, (A) western blotting was performed to analyze β-catenin, c-Myc and cyclin D1. α-tubulin was used as a control; Images are representative of three independent experiments that showed similar results. Grayscale scan analysis of western blot bands were shown in right panel of graph. *p<0.05 and **p<0.01 compared to the cells only transfected with control siRNA. ^##^ *p*<0.01 compared to the cells transfected with β-catenin siRNA along with *p,p′*-DDE treatment. (B) Cell viability and (C) proliferation rate were analyzed by MTT and cell number assay. Values are percent as the mean ± SD of three independent experiments. **p<0.01 compared to control.

**Figure 4:**
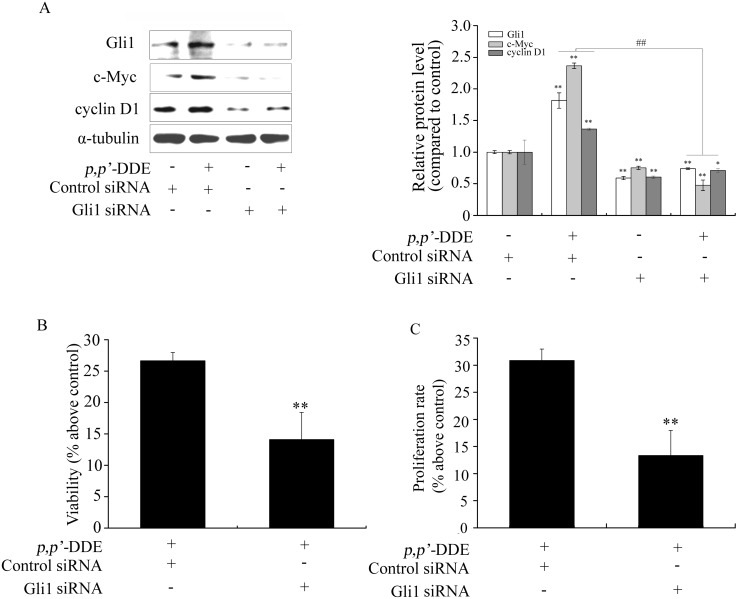
Inhibition of Hedgehog/Gli1 signaling blocks *p,p′*-DDE-induced DLD1 cell proliferation. After DLD1 cells were transfected with control siRNA or Gli1 siRNA, followed by treating with *p,p′*-DDE (10^−9 ^M) for 96 h, (A) Protein levels of Gli1, c-Myc and cyclin D1 were examined by western blotting. α-tubulin was used as a control. Grayscale scan analysis of western blot bands from three independent experiments were shown in right panel of graph. *p<0.05 and **p<0.01 compared to the cells only transfected with control siRNA. ^##^ *p*<0.01 compared to the cells transfected with Gli1 siRNA along with *p,p′*-DDE treatment. (B) Cell viability and (C) proliferation rate were analyzed by MTT and cell number assays. The results are percent as the mean ± SD of three independent experiments. **p<0.01 compared to control.

To further examine the role of Wnt/β-catenin or Hedgehog/Gli1 signaling in *p,p′-*DDE-induced colorectal adenocarcinoma cell proliferation. β-catenin or Gli1 siRNA was used to reduce β-catenin or Gli1 levels and thereby inhibited Wnt/β-catenin or Hedgehog/Gli1 pathway in DLD1 cells. Fig. 3 showed that β-catenin siRNA blocked *p,p′*-DDE-induced DLD1 cell proliferation as well as overexpression of c-Myc and cyclin D1. Similar to the impact of β-catenin siRNA, *p,p′*-DDE-induced DLD1 cell proliferation and overexpression of c-Myc and cyclin D1 were inhibited by Gli1 siRNA (Fig. 4). These results suggested both Wnt/β-catenin and Hedgehog/Gli1 signalings play important roles in *p,p′*-DDE-induced colorectal adenocarcinoma cell proliferation.

### Oxidative stress plays a vital role in *p,p′*-DDE-induced colorectal adenocarcinoma cell proliferation

**Figure 5:**
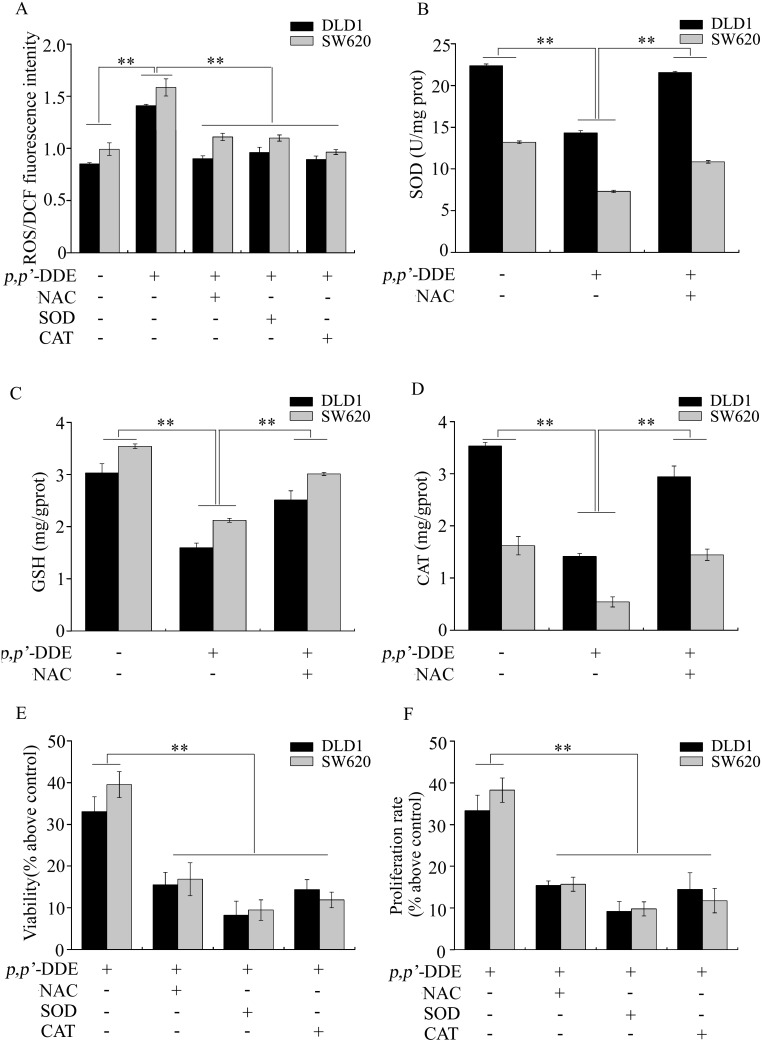
Oxidative stress plays a vital role in *p,p′*-DDE-induced colorectal adenocarcinoma cell proliferation. After DLD1 or SW620 cells were treated with *p,p′*-DDE (10^−9 ^M) alone or co-treated with NAC (10^−3 ^M), SOD (100 U/ml) or CAT (500 U/ml) for 96 h, (A) ROS levels, (B) SOD activity, (C) GSH content, (D) CAT activity, (E) cell viability and (F) proliferation rate were analyzed. The results were the mean ± SD of three independent experiments. **p<0.01 compared to the cells treated with 10^−9 ^M *p,p′*-DDE.

**Figure 6:**
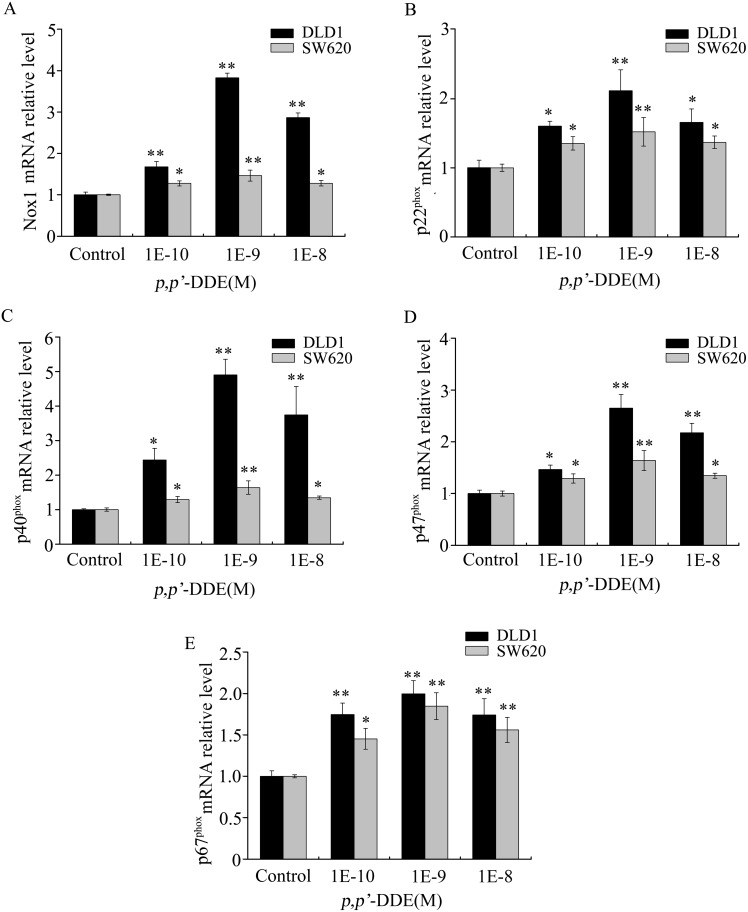
Effects of *p,p′*-DDE on Nox1, p22^phox^, p40^phox^, p47^phox^ and p67^phox^ expression in colorectal adenocarcinoma cells. After DLD1 or SW620 cells were exposed to *p,p′*-DDE (10^−10^, 10^−9^, 10^−8 ^M) for 96 h, the levels of Nox1 (A), p22^phox^ (B), p40^phox^ (C), p47^phox^ (D) and p67^phox^ (E) mRNA expression were determined by quantitative real-time PCR and normalized to control mRNA. The data were acquired from three biologically independent experiments. Values shown were given as the ± SD. *p<0.05 and **p<0.01 compared to control.

Next, we evaluated the components of oxidative stress in *p,p′*-DDE treated cells. As shown in Fig. 5A, *p,p′*-DDE exposure induced 1.7 and 1.6-fold increase in ROS levels in DLD1 and SW620 cells respectively, compared to the negative control. SOD activities were reduced 1.6 and 1.8-fold in *p,p′*-DDE-stimulated DLD1 and SW620 cells, respectively (Fig. 5B). We also found that both GSH content and CAT activity were reduced in *p,p′*-DDE-stimulated cells (Fig. 5C and D). GSH content and CAT activities were reduced 1.9 and 2.5-fold in *p,p′*-DDE-treated DLD1 cells (Fig. 5C and D). In SW620 cells, *p,p′*-DDE led to 1.7 and 3.0-fold decrease in GSH content and CAT activities (Fig. 5C and D). These results indicated that *p,p′*-DDE exposure stimulated oxidative stress in colorectal adenocarcinoma cells. Nicotinamide adenine dinucleotide phosphate (NADPH) oxidases are key enzymes responsible for ROS generation [39]. We examined the effects of *p,p′*-DDE on the expression of NADPH oxidase subunits, including Nox1, p22^phox^, p40^phox^, p47^phox^ and p67^phox^. As shown in Fig. 6, *p,p′*-DDE treatment increased mRNA levels of Nox1, p22^phox^, p40^phox^, p47^phox^ and p67^phox^ in DLD1 or SW620 cells. These results suggested that activation of NADPH oxidase is involved in *p,p′*-DDE-induced ROS generation in colorectal adenocarcinoma cells.

To further determine the role of oxidative stress in *p,p′*-DDE-induced colorectal adenocarcinoma cell proliferation, ROS inhibitor NAC, antioxidant enzyme SOD or CAT was applied to reduce oxidative stress. As shown in Fig. 5, *p,p′*-DDE-induced oxidative stress was significantly reduced after NAC, SOD or CAT treatment (Fig. 5A–D). In addition, *p,p′*-DDE-induced adenocarcinoma cell viability and proliferation were decreased by NAC, SOD or CAT (Fig. 5E and F). Above results suggested that oxidative stress plays a vital role in *p,p′*-DDE-induced colorectal adenocarcinoma cell proliferation.

### Inhibition of oxidative stress decreases *p,p′*-DDE induced Wnt/β-catenin and Hedgehog/Gli1 signalings activation

**Figure 7:**
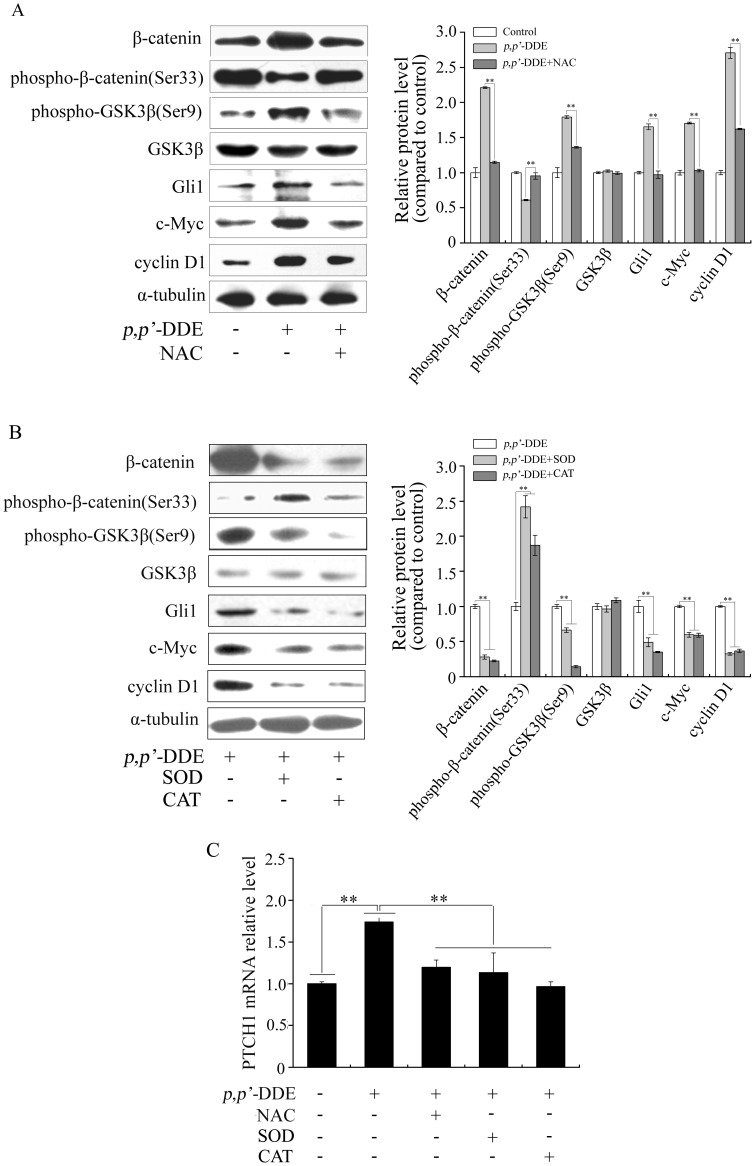
The antioxidants inhibit *p,p′*-DDE-induced Wnt/β-catenin and Hedgehog/Gli1 signalings activation in DLD1 cells. After DLD1 cells were treated with *p,p′*-DDE (10^−9 ^M) alone or co-treated with NAC (10^−3 ^M) (A), SOD (100 U/ml) or CAT (500 U/ml) (B) for 96 h, western blotting was performed to analyzed β-catenin, phospho-β-catenin (Ser33), phospho-GSK3β (Ser9), GSK3β, Gli1, c-Myc and cyclin D1. α-tubulin was used as the loading control. Grayscale scan analysis of western blot bands were shown in right panel of graph. (C) mRNA expression of PTCH1 was quantified by quantitative real-time PCR analysis. Relative mRNA levels were normalized with control mRNA. In (A), (B) and (C), value shown was given as the ± SD and acquired from three biologically independent experiments. **p<0.01 compared to the cells treated with 10^−9 ^M *p,p′*-DDE.

Accumulated evidences showed both Wnt/β-catenin and Hedgehog/Gli1 signalings could been stimulated by oxidative stress [36], [40]–[45]. We speculated that both Wnt/β-catenin and Hedgehog/Gli1 signalings could been mediated by *p,p′*-DDE through oxidative stress. To verify this hypothesis, cells were treated with *p,p′*-DDE combined with NAC, SOD or CAT for 96 h. The results showed treatment with NAC, SOD or CAT prevented *p,p′*-DDE-induced the up regulation of β-catenin, phospho-GSK3β (Ser9), Gli1, PATCH1, c-Myc and cyclin D1 as well as down regulation of phospho-β-catenin (Ser33) (Fig. 7 and S3). These results suggested that both Wnt/β-catenin and Hedgehog/Gli1 signalings were mediated by oxidative stress in *p,p′*-DDE-stimulated cells.

## Discussion

**Figure 8:**
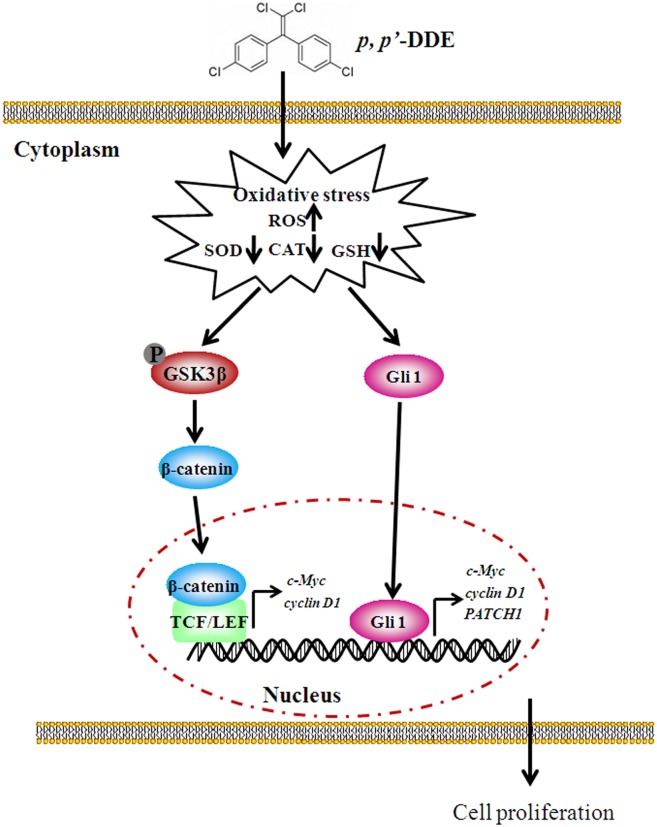
Proposed diagram of *p,p′*-DDE-induced colorectal adenocarcinoma cell proliferation. At low concentrations, *p,p′*-DDE promotes colorectal adenocarcinoma cell proliferation through c-Myc and cyclin D1 overexpression resulted from the stimulation of Wnt/β-catenin and Hedgehog/Gli1 signalings, which were mediated by oxidative stress.

*p,p′*-DDE, the major metabolite of DDT, is a hazardous persistent chemicals presented in environment, and attracts extensive public attentions on its adverse health effects. In the present study, we provide experimental supports for *p,p′*-DDE promoting the development of colorectal cancers through inducing cancer cell proliferation. The possible role of *p,p′*-DDE in inducing colorectal cell proliferation is summarized in Fig. 8. At low concentrations, *p,p′*-DDE exposure stimulates oxidative stress, followed by activating Wnt/β-catenin and Hedgehog/Gli1 signalings. Abnormal activation of two-crucial pathways results in overexpression of downstream target proteins c-Myc and cyclin D1 and thus induces the proliferation of colorectal cancer cells.

Our results indicated that *p,p′*-DDE exposure at low concentrations (10^−12^ to 10^−7 ^M) significantly promoted colorectal adenocarcinoma cell proliferation. However, high concentration of *p,p′*-DDE (10^−4 ^M) significantly inhibited cell viability and viable cell counts. These results suggested that low concentrations of *p,p′*-DDE present the inducibility on colorectal adenocarcinoma cell proliferation. Previous observations found high concentrations of *p,p′*-DDE (>3×10^−5 ^M) induced rat sertoli cell apoptosis [34], [46], which were consistent with our results.

It has been known that aberrant Wnt/β-catenin signaling is discovered in about 80% colorectal cancers [25], [26]. In colorectal cancers, mutations in components of Wnt/β-catenin pathways such as adenomatous polyposis coli (APC), Axin and β-catenin are common and render β-catenin difficult for degradation by cellular proteosomes [45]. GSK3β is a serine/threonine protein kinase and is inhibited by phosphorylation at Ser 9 residual [47]. GSK3β destabilizes β-catenin by phosphorylating it at Ser33, 37 and Thr41 [48]. Our present findings revealed that total and nuclear β-catenin were significantly increased in *p,p′*-DDE-stimulated cells. *p,p′*-DDE exposure also led to unregulated phosphorylation of GSK-3β at Ser 9 along with reduced phosphorylation of β-catenin at Ser 33. These results suggested GSK-3β activity was inhibited and thereby led to β-catenin stabilization during *p,p′*-DDE-induced colon cancer cell proliferation. Hedgehog/Gli1 signaling is implicated to be important in the maintenance of colon tumor [27], [28], [49]. Hedgehog/Gli1 signaling related components, PTCH1 and Gli1, are over-expressed in colon tumor [27], [50]–[52]. Our results showed that *p,p′*-DDE exposure markedly upregulated total and nucleus Gli1, as well as PTCH1 mRNA levels. PTCH1 is not only the component of Hedgehog/Gli1 signaling but also regulated by Gli1 [53]. It is likely that increased Gli1 induced by *p,p′*-DDE elevated PTCH1 mRNA levels. It has been reported that both β-catenin and Gli1 mediate proto-oncogenes including *c-myc* and *cyclin D1* [29], [32], [33]. This study found both c-myc and cyclin D1 were increased after *p,p′*-DDE exposure, and knockdown of β-catenin or Gli1 prevented *p,p′*-DDE-induced generation. These results indicated c-myc and cyclin D1 were regulated by Wnt/β-catenin and Hedgehog/Gli1 signalings in *p,p′*-DDE-stimulated colorectal adenocarcinoma cells.

*p,p′*-DDE-induced toxicity is associated with oxidative stress. *p,p′*-DDE can induce apoptosis by elevating ROS production [46], [54]. In addition, *p,p′*-DDE exposure also could result in increased MDA (a product of lipid peroxidation) as well as decreased SOD and GSH-Px activities [46], [55]. In this study, we found elevated ROS production, lower GSH content, SOD and CAT activities in *p,p′*-DDE-treated colorectal adenocarcinoma cells. These results were consistent with previous investigation. NADPH oxidase is a membrane-localized enzyme responsible for ROS generation. NADPH oxidase utilize NADPH as an electron donor to generate superoxide(O_2_^−^) [56]. Various NADPH oxidase isoforms (Nox1-Nox7) have been found. Nox1 is highly expressed in colonic epithelial cells and integrates Wnt/β-catenin and Notch1 signals to control colon progenitor cell proliferation and fate [57]. In human colon cancers, Nox1 is overexpressed and correlates with ROS–dependent cancer invasion [58], [59]. It has been reported that NADPH oxidase activation is involved in arsenic-induced cell transformation and tumorigenesis in human colorectal adenocarcinoma cells [41]. Our results showed that *p,p′*-DDE increased expression of Nox1, p22^phox^, p40^phox^, p47^phox^ and p67^phox^. These observation suggested that NADPH oxidase activation plays crucial role in *p,p′*-DDE induced oxidative stress in colorectal adenocarcinoma cells. Many investigators have suggested the involvement of oxidative stress in toxicant-induced carcinogenesis. For example, cadmium, arsenic and chromium could promote tumorigenesis via an ROS-mediated pathway [36], [41], [42]. Our previous study demonstrated that *p,p′*-DDT, precursor of *p,p′*-DDE induced colorectal cancer growth through oxidative stress-mediated pathways [37]. To verify the involvement of oxidative stress in *p,p′*-DDE-induced colorectal adenocarcinoma cell proliferation, cells were co-exposured to both *p,p′*-DDE and NAC, SOD or CAT. Treatment with NAC, SOD or CAT reduced *p,p′*-DDE-induced cell proliferation, which indicated that oxidative stress plays important role in *p,p′*-DDE-induced colorectal adenocarcinoma cell proliferation.

Our study found that oxidative stress, Wnt/β-catenin and Hedgehog/Gli1 signalings were involved in *p,p′*-DDE-induced colorectal adenocarcinoma cell proliferation. Many studies found oxidative stress-mediated signalings include both Wnt/β-catenin and Hedgehog/Gli1 signalings. Zhang *et al* reported that arsenic induced colorectal adenocarcinoma cell transformation and tumorigeneis through ROS-mediated Wnt/β-catenin signaling [41]. ROS regulated arsenic and chromium-induced tumorigenesis via Wnt/β-catenin signaling in a mouse colitis-associated colorectal cancer model [36]. Hedgehog/Gli1 signaling was activated under H_2_O_2_-induced oxidative stress in cultured astrocytes [60]. To clarify whether both Wnt/β-catenin and Hedgehog/Gli1 signalings are mediated through oxidative stress in *p,p′*-DDE-stimulated cells, ROS inhibitor NAC, antioxidant enzyme SOD or CAT was employed to reduce oxidative stress, followed by assessing both Wnt/β-catenin and Hedgehog/Gli1 signalings. Treatment of NAC, SOD or CAT not only attenuated the induction of β-catenin, phospho-GSK3β (Ser9), Gli1, PATCH1, c-Myc and cyclin D1, but also prevented the downregulation of phospho-β-catenin (Ser33). These results strongly suggested that oxidative stress activated both Wnt/β-catenin and Hedgehog/Gli1 signalings.

In conclusion, our study demonstrates for the first time that *p,p′*-DDE exposure stimulates proliferation of colorectal cell proliferation, leading to colorectal cancer progression. *In vitro* exposure of *p,p′*-DDE can enhance oxidative stress, then induce activation in Wnt/β-catenin and Hedgehog/Gli1 signalings. These effects result in increased expression of downstream target proteins c-Myc and cyclin D1 and thereby induce colorectal cell proliferation. The present study provides important data for further study of cancer development resulting from xenobiotic compounds.

## Supporting Information

Figure S1 **Effects of high concentrations of** ***p,p′*** **-DDE on colorectal adenocarcinoma** **cell proliferation.** After DLD1 or SW620 cells were exposed to *p,p′*-DDE (10^−5^ and 10^−4 ^M) for 96 h, inhibition rate(%) were determined using MTT (A) and cell number assays (B), respectively. Values are percent as the mean ± SD of three independent experiments. **p<0.01 compared to control cells.
(TIF)

Figure S2 ***p,p′*** **-DDE upregulates Wnt/β-catenin and Hedgehog/Gli1 signalings in SW620 cells.** After SW620 cells were treated with *p,p′*-DDE (10^−10^, 10^−9^, 10^−8 ^M) for 96 h, (A) western blotting was performed to analyze β-catenin, phospho-β-catenin (Ser33), phospho-GSK3β (Ser9), GSK3β, Gli1, c-Myc, cyclin D1 and PCNA levels. α-tubulin was used as the loading control. (B) Quantitative real-time PCR was performed to determine the level of PTCH1 mRNA expression. Relative mRNA levels were normalized with control mRNA. Values shown were given as the ± SD and acquired from three independent experiments. **p<0.01 compared to control.
(TIF)

Figure S3 **Effects of antioxidants on** ***p,p′*** **-DDE-induced Wnt/β-catenin and Hedgehog/Gli1 signalings activation in SW620 cells.** (A) After SW620 cells were treated with *p,p′*-DDE (10^−9 ^M) alone or co-treated with NAC (10^−3 ^M), SOD (100 U/ml) or CAT (500 U/ml) for 96 h, western blotting was performed to analyzed β-catenin, phospho-β-catenin (Ser33), phospho-GSK3β (Ser9), GSK3β, Gli1, c-Myc and cyclin D1. α-tubulin was used as the loading control. (B) mRNA expression of PTCH1 was determined by quantitative real-time PCR analysis and normalized to control mRNA. Values were presented as the ± SD and acquired from three independent experiments. **p<0.01 compared to the cells treated with 10^−9 ^M *p,p′*-DDE.
(TIF)
