# Supplementary figures and images for: p, p′-Dichlorodiphenyldichloroethylene Induces Colorectal Adenocarcinoma Cell Proliferation through Oxidative Stress
Source: PLoS One. 2014 Nov 11;9(11):e112700. doi: 10.1371/journal.pone.0112700 (PMC4227882; doi:10.1371/journal.pone.0112700)

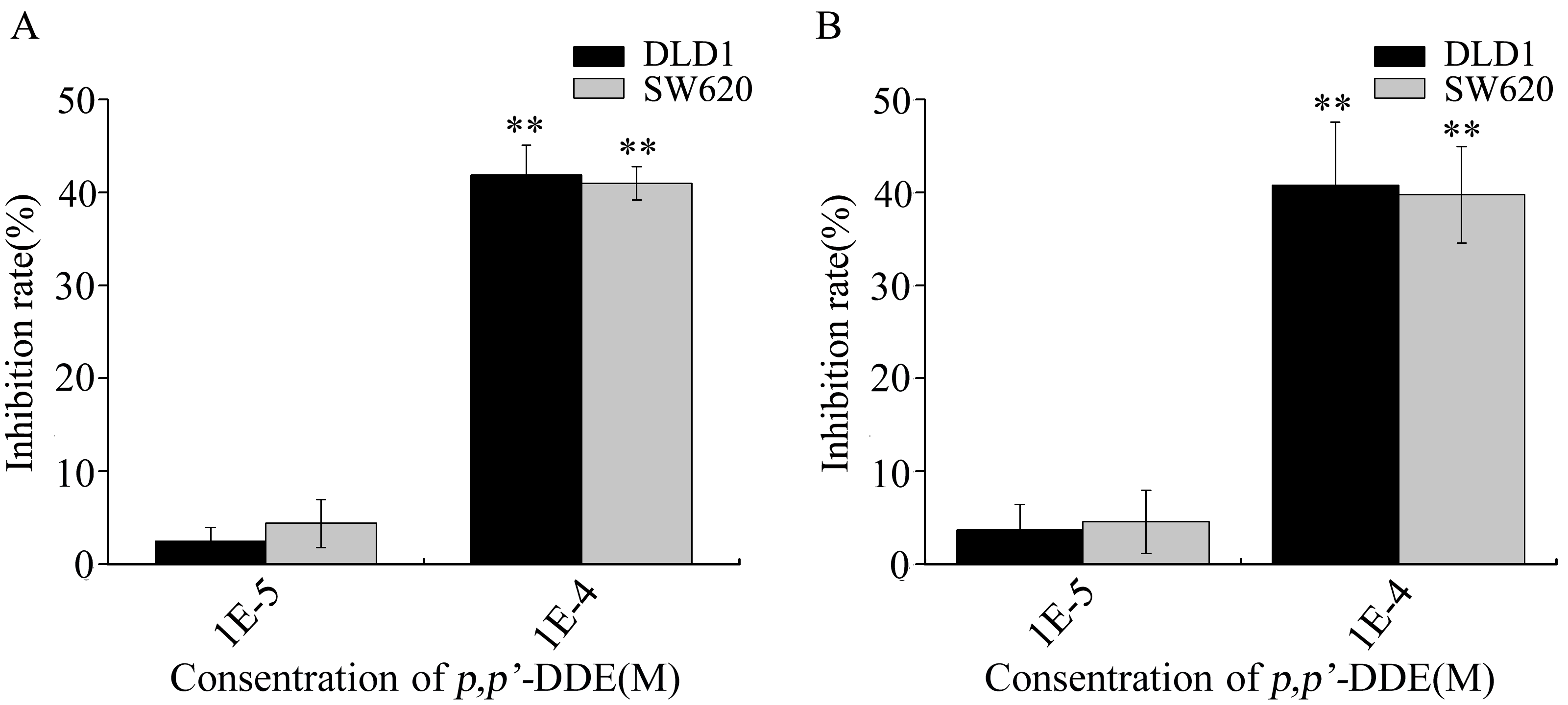

Supplement: Figure S1 — Effects of high concentrations of p,p′ -DDE on colorectal adenocarcinoma cell proliferation. After DLD1 or SW620 cells were exposed to p,p′-DDE (10−5 and 10−4 M) for 96 h, inhibition rate(%) were determined using MTT (A) and cell number assays (B), respectively. Values are percent as the mean ± SD of three independent experiments. **p<0.01 compared to control cells. (TIF) [file pone.0112700.s001.tif]

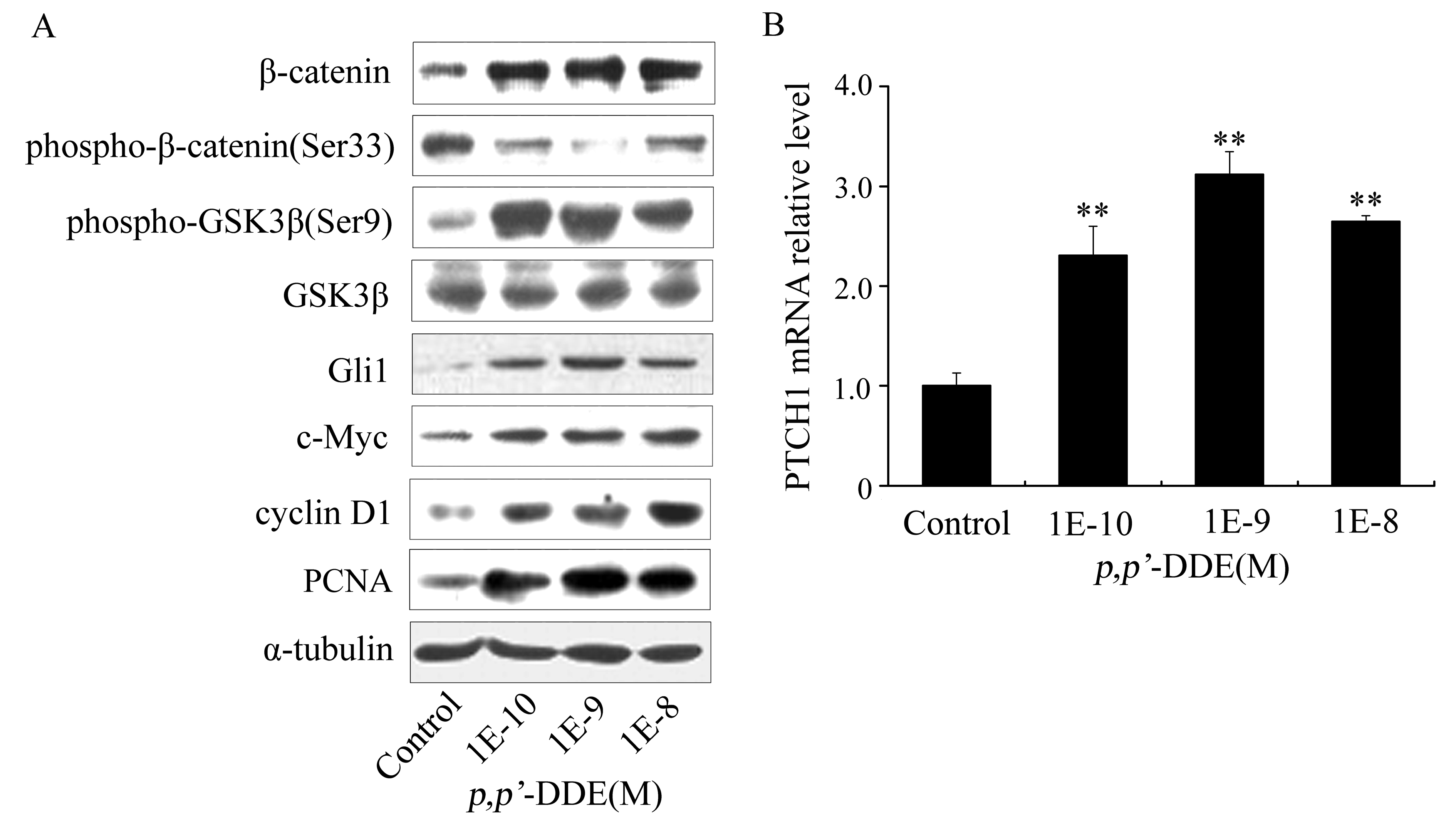

Supplement: Figure S2 — p,p′ -DDE upregulates Wnt/β-catenin and Hedgehog/Gli1 signalings in SW620 cells. After SW620 cells were treated with p,p′-DDE (10−10, 10−9, 10−8 M) for 96 h, (A) western blotting was performed to analyze β-catenin, phospho-β-catenin (Ser33), phospho-GSK3β (Ser9), GSK3β, Gli1, c-Myc, cyclin D1 and PCNA levels. α-tubulin was used as the loading control. (B) Quantitative real-time PCR was performed to determine the level of PTCH1 mRNA expression. Relative mRNA levels were normalized with control mRNA. Values shown were given as the ± SD and acquired from three independent experiments. **p<0.01 compared to control. (TIF) [file pone.0112700.s002.tif]

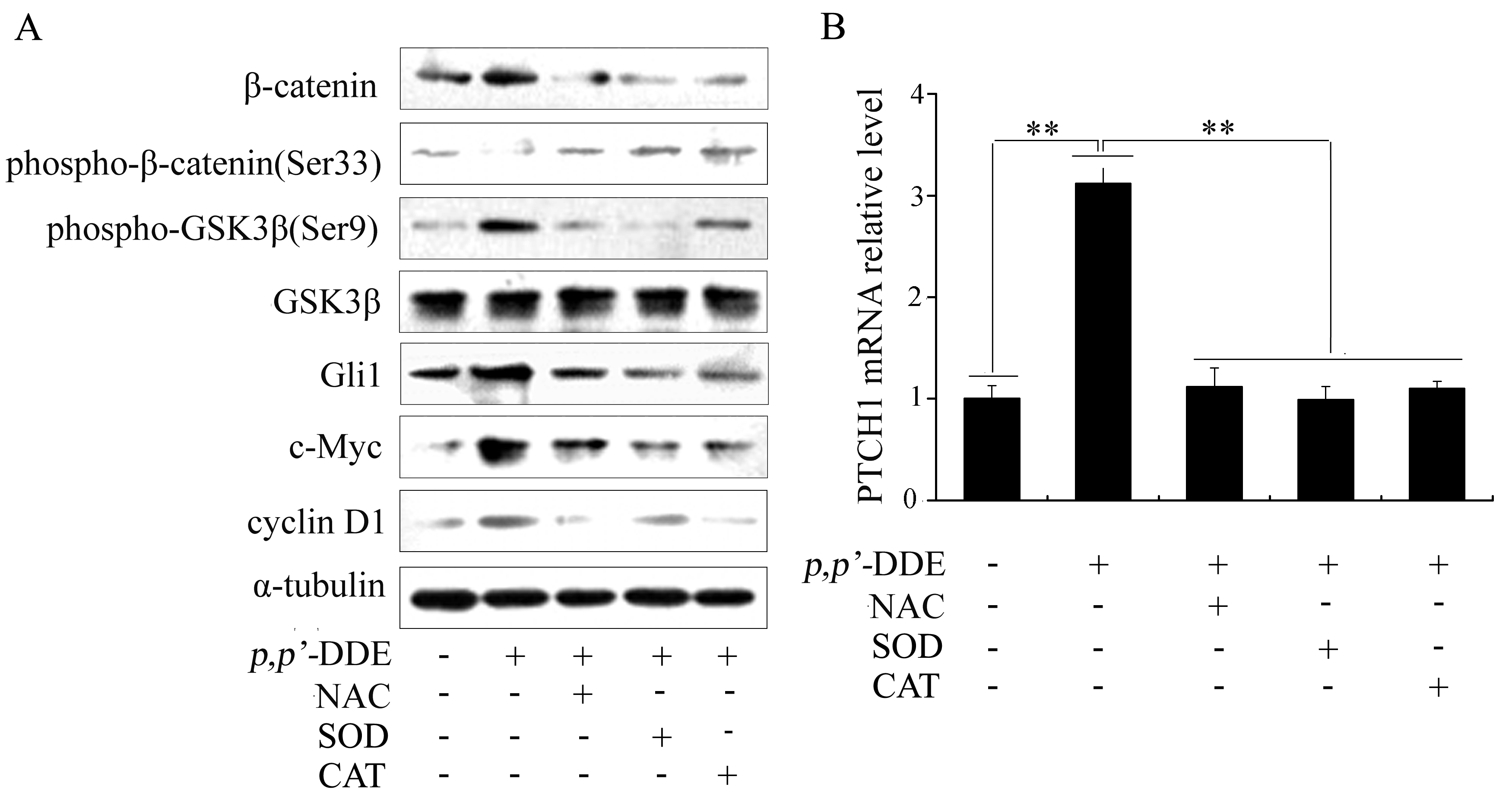

Supplement: Figure S3 — Effects of antioxidants on p,p′ -DDE-induced Wnt/β-catenin and Hedgehog/Gli1 signalings activation in SW620 cells. (A) After SW620 cells were treated with p,p′-DDE (10−9 M) alone or co-treated with NAC (10−3 M), SOD (100 U/ml) or CAT (500 U/ml) for 96 h, western blotting was performed to analyzed β-catenin, phospho-β-catenin (Ser33), phospho-GSK3β (Ser9), GSK3β, Gli1, c-Myc and cyclin D1. α-tubulin was used as the loading control. (B) mRNA expression of PTCH1 was determined by quantitative real-time PCR analysis and normalized to control mRNA. Values were presented as the ± SD and acquired from three independent experiments. **p<0.01 compared to the cells treated with 10−9 M p,p′-DDE. (TIF) [file pone.0112700.s003.tif]
